# Effect of Phenolic Compounds on Human Health

**DOI:** 10.3390/nu13113922

**Published:** 2021-11-01

**Authors:** Elena González-Burgos, M. Pilar Gómez-Serranillos

**Affiliations:** Department of Pharmacology, Pharmacognosy and Botany, Faculty of Pharmacy, Universidad Complutense de Madrid, 28040 Madrid, Spain; pserra@ucm.es

This book, based on a Special Issue of *Nutrients*, contains a total of 12 papers (8 original research and 4 reviews) on the effect of phenolic compounds on human health. The consumption of exogenous medicinal plants and food rich in phenolic compounds represents a promising therapeutically to prevent many chronic diseases, including diabetes, cardiovascular diseases, cancer, and neurodegenerative diseases, among others.

The original articles include three in vitro studies, one in vivo work, and four clinical trials. Regarding in vitro studies, two of them study the effect of polyphenols on obesity. Hence, *Inula Britannica* flower aqueous extract, which is rich in phenolic compounds, has shown to inhibit adipogenesis through modulation of mitotic clonal expansion and the extracellular signal-regulated kinase 1/2 and Akt signaling pathways in 3T3-L1 adipocytes [1]. Likewise, in another in vitro study, it has been shown that anthocyanin and non-anthocyanin polyphenol fractions from lingonberry fruit could help prevent and treat obesity and endothelial dysfunction by reducing oxidative stress and inflammation through enhancing antioxidant enzyme expression and inhibiting ROS production and pro-inflammatory genes expression and adhesion molecules [2]. In addition to these anti-obesity effects of polyphenols, their neuroprotective role has been demonstrated in other work. Particularly, methanol extracts of *Moringa oleifera* leaf powder, rich in polyphenols with potent antioxidant properties, protected human neuroblastoma cells from H_2_O_2_-induced oxidative stress. Pretreatments with concentration of 25 µg/mL of moringa extracts reduced reactive oxygen production and lipid peroxidation, as well as increasing the reduced glutathione content and antioxidant enzymatic activity. Moreover, this study revealed that moringa can act at the mitochondrial level by regulating calcium levels and increasing mitochondrial membrane potential [3].

The only in vivo study included in this book covers the preventive role of grape seed-derived procyanidins (GSPE) (500 mg/kg) in age-related processes in female rats, such as pancreas dysfunction and tumor development [4].

Finally, four clinical trials are compiled. Two of them have shown the benefits of polyphenols in overweight and obese people. Hence, supplementation with a polyphenol-rich ingredient (900 mg/day for 16-week) improved the physical and mental health of overweight and obese volunteers [5]. Moreover, a combination of trans-resveratrol and hesperetin (tRES-HESP, 90–120 mg) reduced dysglycemia, blood pressure, vascular inflammation, and dyslipidemia in healthy, overweight, and obese subjects. These beneficial effects are related to its ability to counteract methylglyoxal accumulation and protein glycation and to decrease TXNIP and TNFα expression. The study also demonstrated that these compounds exert their effects synergistically [6]. In other work, it has been seen that a diet rich in polyphenols in ultra-endurance athletes enhance both exercise performance and post-exercise recovery for balancing redox balance [7]. Furthermore, this book also included a cross-sectional study to investigate the multidirectional interactions between TAS2R genotype [TAS2R4 gene (rs2233998 and rs2234001); TAS2R5 gene (rs2227264)], epicatechin intake, and body mass index (BMI), together in an elderly cohort. This study demonstrated that there is not an association between epicatechin intake and BMI and TAS2R genotype [8].

Regarding the reviews, two general papers on the effect of dietary polyphenols as immunomodulatory and liver protector agents have been included [9,10], and two more specific papers focused on the benefits of hop components in beer, rich in flavonoids, as neuroprotective agents, based on its antioxidant and anti-inflammatory activities, and the benefits of citrus flavonoids in diabetes [11,12].

We believe that this collection includes a summary of current studies on the benefits of polyphenols and of great value for future research in this area.
